# Development and Assessment of a Color-Variable Chlorine Dioxide Slow-Releasing Card for Litchi Preservation

**DOI:** 10.3390/foods14010136

**Published:** 2025-01-06

**Authors:** Li Guo, Guang Wu, Qingchun Yin, Lin Luo, Hao Deng

**Affiliations:** 1 Guangdong Provincial Key Laboratory of Food Quality and Safety, College of Food Science, South China Agricultural University, Guangzhou 510641, China; nevertheless25@163.com; 2 Key Laboratory of Tropical Fruit and Vegetable Cold-Chain of Hainan Province, Institute of Agro-Products of Processing and Design, Hainan Academy of Agricultural Sciences, Haikou 571100, China; wuguang@hnaas.org.cn; 3 Key Laboratory of Genetic Resources Evaluation and Utilization of Tropical Fruits and Vegetables (Co-Construction by Ministry and Province), Ministry of Agriculture and Rural Affairs, Haikou 571100, China; 4 Sanya Institute of Hainan Academy of Agricultural Sciences, Sanya 572019, China; 5 Key Laboratory of Tropical Fruits and Vegetables Quality and Safety, State Administration for Market Regulation, Institute of Food Testing, Hainan Academy of Inspection and Testing, Haikou 570314, China; yinqingchun@163.com

**Keywords:** ClO_2_ slow-releasing card, color, litchi, development, assessment

## Abstract

Chlorine dioxide (ClO_2_) gas has attracted considerable attention due to its safety and efficiency. In this study, we successfully developed a color-variable ClO_2_ slow-releasing card for postharvest litchi. The optimal ClO_2_ slow-releasing card was prepared as follows: Card A was soaked in 2.5 mol/L NaClO_2_ and 0.3 mol/L CaCl_2_. Card B was soaked in 1 mol/L oxalic acid, 0.3 mol/L CaCl_2_, and natural pigment. Finally, cards A and B were dried and adhered using 60% gelatin. The ClO_2_ releasing time of the card was more than 120 h at 5–25 °C, and it could change color from dark yellow to white. The qualities of 3 kg litchi treated with no pieces (CK), half a piece (T1), one piece (T2), two pieces (T3), and three pieces (T4) of ClO_2_ slow-releasing card were compared. The results showed that litchi of T1 had significantly (*p* < 0.05) higher L* and a* values but a lower respiration rate and relative conductivity than CK after 7 days of storage, showing the best pulp qualities and pericarp color. Further correlation analyses revealed a significant positive correlation (R^2^ = 0.971) between Cya-3-O-gal-1 and a*, indicating a sharp decline in Cya-3-O-gal-1 and strong pericarp browning in CK. On the contrary, the low-releasing ClO_2_ of T1 effectively inhibited Cya-3-O-gal-1 degradation. This could be one of the reasons for the superior pericarp color of T1. This study provides a visual, efficient, and economical solution for postharvest litchi.

## 1. Introduction

Litchi (*Litchi chinensis* Sonn.), belonging to the Sapindaceae family, is a tropical and subtropical fruit native to China [1,2]. Its harvesting typically occurs during the warm months from April to June. A number of biochemical and physiological changes and microbial invasions take place after the fruit is harvested, which affect the quality of the fruit [3]. Also, the postharvest storage and transport process of litchi often lead to fruit deterioration and cause certain economic losses [4,5,6]. Intense postharvest respiration and high levels of sugars can easily lead to fruit browning, deterioration, and microbial infection [1,7]. In addition, litchi is rich in anthocyanins [8,9], and the degradation process, catalyzed by polyphenol oxidase (PPO) and peroxidase (POD) enzymes, can expedite the browning of the pericarp within one or two days [10,11,12,13,14]. Therefore, microorganism infection and pericarp browning are crucial concerns regarding postharvest litchi.

Chlorine dioxide (ClO_2_), as an efficient and safe broad-spectrum antimicrobial preservative, has been widely used in postharvest litchi and other tropical fruits [15,16,17]. Unlike sulfur dioxide (SO_2_), ClO_2_ is considered safer due to its minimal production of toxic chlorination by-products in fruits [18]. It was proven to reduce external pathogens and extend the shelf life of fruit even at low concentrations [19]. However, the application of ClO_2_ in fruit preservation is limited by its instability and the necessity of on-site production equipment.

Recently, researchers have increasingly focused on the instrument-free preservation method utilizing ClO_2_. Wu et al. devised a preservative utilizing a carboxymethyl chitosan–citric acid complex (CMC-CA) that slowly releases ClO_2_, specifically tailored for longan [20]. The reaction for producing stable ClO_2_, which occurs between sodium chlorite (NaClO_2_) and carboxymethyl chitosan-citric acid (CMC-CA), can be initiated by the natural humidity present in longan fruit during its preservation. Ren et al. utilized EC/SA-NaClO_2_ microcapsules and a HCl-containing polyvinyl alcohol (PVA) solution to create an antimicrobial film [19]. The reaction between HCl and NaClO_2_ within the microcapsules was triggered by water vapor to release ClO_2_. Similarly, Liu et al. prepared a PVA film that could release ClO_2_ automatically to kill pathogens and extend the shelf life of litchi [21]. However, a burst release of ClO_2_ is a great challenge when ClO_2_-releasing powder or film is in contact with condensed water droplets. In addition, direct contact of ClO_2_ preservatives with fruit increases the risk of chemical residues. ClO_2_ in low concentrations is a colorless, odorless gas. Determining how to visualize the ClO_2_-producing reaction is another huge challenge. To date, there is still limited research on instrument-free ClO_2_ preservative products with a visual indicator.

In this study, we aimed to devise a novel card capable of slowly releasing ClO_2_ whereby the color of the card can change automatically according to ClO_2_ release. This card can absorb moisture from air to trigger the reaction of releasing ClO_2_. Then, the basic qualities and crucial anthocyanins were determined to evaluate the preservative effects of this card on postharvest litchi. This study could offer a visual, efficient, and low-cost solution for postharvest litchi.

## 2. Materials and Methods

### 2.1. Plant Material and Chemical Reagent

Litchi was purchased from Haikou South and North Fruit Market (Haikou, Hainan, China). The cultivar of the litchi was ‘Nuomici’, and it was of commercial maturity. All the fruits underwent rigorous selection, were uniform in size and devoid of pests and diseases, and lacked any mechanical injuries.

Sodium chlorite (purity ≥ 99.4%) was purchased from Guangdong Wenglong Chemical Reagent Co., Ltd. (Shaoguan, China). Oxalic acid (Purity ≥ 99.4%) was purchased from Tianjin Huasheng Chemical Reagent Co., Ltd. (Tianjin, China). Potassium iodide (Purity ≥ 99.0%) was purchased from Tianjin Huasheng Chemical Reagent Co., Ltd. (Tianjin, China). Sodium thiosulfate (Purity ≥ 99.0%) was purchased from Tianjin Zhiyuan Chemical Reagent Co., Ltd. (Tianjin, China). The other reagents were analytical-grade reagents.

### 2.2. Preparation and Optimization of ClO_2_ Slow-Releasing Card

ClO_2_ slow-releasing card: two paper cards (40 × 40 mm^2^) were adhered together by a press machine (SK-50H, SECCO Automation Co., Ltd., Zhuhai, China) after immersion in four main components, including NaClO_2_, acid, an absorbent, and natural pigment. Card A was soaked in NaClO_2_ and absorbent aqueous solution for 30 min. Similarly, card B was soaked in acid, natural pigment, and absorbent aqueous solution for 30 min. The main composition of the ClO_2_ slow-releasing card including NaClO_2_, three acids (oxalic acid, tartaric acid, and citric acid), three absorbents (NaSO_4_, MgSO_4_, and CaCl_2_), and three adhesives (gelatin, sodium carboxymethyl cellulose, and sodium alginate) was first assessed. The concentrations were further refined through L16(4^4^) orthogonal experiments, as detailed in Table S1. Based on the total amount of ClO_2_ released within 4 days, the optimal formulation of the slow-release ClO_2_ preservation card was obtained.

### 2.3. Color Indicator of ClO_2_ Slow-Releasing Card

Natural pigment was purchased from Guangzhou Nature Food Co., Ltd. (Guangzhou, China). The main components of this pigment were 64.5% glycerin, 5% monascus red, 2.8% carmine, and 27.7% water. β-cyclodextrin was added to enhance the stability of the coloring. The ratios of pigment to β-cyclodextrin were 15:1, 15:5, and 15:10, respectively. The L* (lightness), a* (redness), and b* (yellowness) values were measured by a spectrophotometer (LS175, Shenzhen Linshang Technology Co., Ltd., Shenzhen, China). Subsequently, the color contribution index (CCI) was calculated using the equation CCI = (1000 × a*)/(L* × b*). The natural pigment was pH-sensitive and could be easily oxidized by released CIO_2_. The greater the change in the CCI value, the more CIO_2_ was released.

### 2.4. Stability Evaluation of ClO_2_ Slow-Releasing Card

For the assessment of the ClO_2_ slow-releasing card’s stability, a single card was inserted into a 0.9 L sealed container, and the ClO_2_ release rate was determined as described in Section 2.4, within 120 h at temperatures ranging from 5 to 25 °C. Humidity was maintained at 95% in constant-temperature and -humidity chambers.

### 2.5. ClO_2_ Release Rate of ClO_2_ Slow-Releasing Card

The measurements were based on Wu et al. [20]. The volume of Na_2_S_2_O_3_ used was recorded for the calculation of the ClO_2_ release rate using formula (1):

ClO_2_ release rate (mg/h) = (0.02 × *V* × 67.5)/12 (1)

where *V* was the volume of Na_2_S_2_O_3_ consumed in mL.

### 2.6. Application of ClO_2_ Slowing-Releasing Card for Litchi Preservation

Five different treatments were established: 0 pieces (CK), 0.5 pieces (T1), 1 piece (T2), 2 pieces (T3), and 3 pieces (T4) of slow-releasing card, each placed in a sealed foam box (6 L) containing 3 kg of litchi. The boxes were kept at 10 °C and a relative humidity level of 95%. L*, a*, b*, TSS, and other indicators of relevance were examined at 1, 3, 5, and 7 days.

### 2.7. Qualities of Stored Litchi

The spectrophotometer (LS175, Shenzhen Linshang Technology Co., Ltd., Shenzhen, China) was utilized to measure the L*, a*, and b* values. The TSS of the litchi were analyzed using a Brix refractometer (AK002C, Dongguan AIOK Electronic Co., Ltd., Dongguan, China). A total of 400 g of litchis of each group were selected and sealed in a 2.5 L glass jar for gas equilibration for 5 min. Then, the respiration rate was determined by a respiration tester (YT-GX10, Shandong Yuntang Science and Technology Co., Ltd., Weifang, China). The relative conductivity of litchi peel was determined using an electrical conductivity meter (TDS-10, Shanghai Lichen Instrument Technology Co., Ltd., Shanghai, China). There were three replications of all the above analyses.

### 2.8. Anthocyanins of Stored Litchi

One gram of peel was mixed with 10 mL of acetonitrile and 2 ceramic homogenizers. The mixture was stirred for 10 min and sonicated for 10 min. After centrifugation at 10,000 r/min for 5 min, 1 mL of the supernatant was transformed and diluted 5 times. The diluted solution was filtered using a membrane (SCAA-104, 0.22 μm; ANPEL, Shanghai, China) before being analyzed via ultra-performance liquid chromatography–tandem mass spectrometry (UPLC-MS/MS).

UPLC: A Thermo Scientific UltiMate 3000 UHPLC (Waltham, MA, USA) system equipped with a chromatographic Phenomenex Luna Omega Polar C18 column (1.6 μm, 2.1 mm × 100 mm) was used for analysis. A total of 5.0 μL sample was loaded into the column and maintained at 30 °C with a 0.3 mL/min flow rate of mobile phase. Mobile phase A was methanol and mobile phase B was water. The mobile phase gradient is shown in Table S2.

MS/MS: The effluent was connected to an AB Sciex QTRAP 4500 mass spectrometer (AB Sciex, Framingham, MA, USA). The detailed MS parameters of 27 anthocyanins are shown in Table S3. The concentration of anthocyanin was calculated using Formula (2):

The concentration of anthocyanin:

(2) $$x=\frac{C\times V\times D}{m\times 1000}$$

where x represents the concentration of anthocyanin, mg/kg; *C* is the sample concentration, ng/mL; *V* is the sample volume, mL; *m* is the sample weight, g; and *D* is the dilution factor.

### 2.9. Statistical Analysis

The dataset was analyzed using SPSS 22 version software (IBM, Armonk, NY, USA), and the means were compared using Duncan’s novel multiple-range test. Statistical significance was set at *p* < 0.05. For both the statistical analysis and graphical representation, Origin Lab’s 2019b version (Origin Lab Inc., Northampton, MA, USA) was utilized.

## 3. Results and Discussion

### 3.1. Optimization of Composition in ClO_2_ Slow-Releasing Card

**Figure 1:**
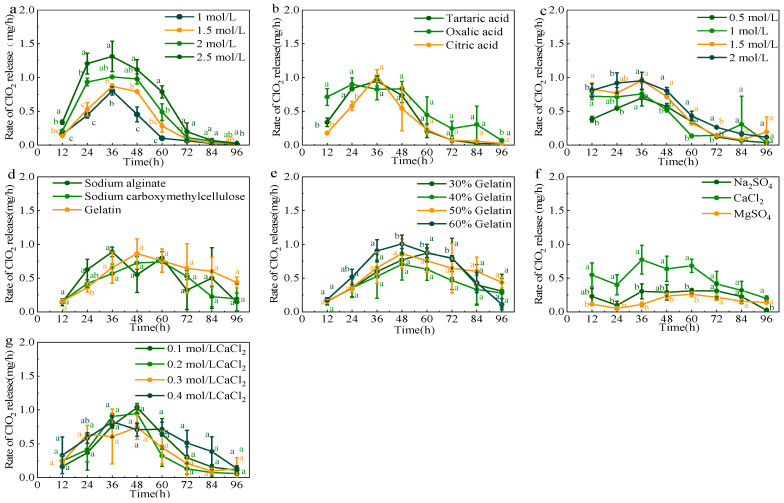
Optimization of NaClO_2_ concentration (**a**), acids (**b**), oxalic acid concentration (**c**), adhesives (**d**), gelatin concentration (**e**), absorbents (**f**), and absorbent concentration (**g**) of the card. Different letters represent significant (*p* < 0.05) changes between groups on the same day.

A single-factor experiment was initially conducted to optimize the composition of the four components in the ClO_2_ slow-releasing card. Subsequently, their concentrations were further optimized using L16(4^4^) orthogonal experiments. As shown in Figure 1b, when citric acid was utilized as the H^+^ provider, a sudden release of ClO_2_ occurred. The ClO_2_ release rate peaked at 1.03 mg/h after 36 h, followed by a rapid decline. Conversely, oxalic acid proved to be the most stable H^+^ provider in 96 h. As shown in Figure 1c, the concentration of oxalic acid was further optimized. An increased concentration of oxalic acid led to a correspondingly higher release rate of ClO_2_. The ClO_2_ release rate reached a maximum of 0.96 mg/h at 24 h and remained the highest throughout the release period when 2 mol/L oxalic acid was added. The ClO_2_ slow-releasing card, which employed gelatin as the adhesive and CaCl_2_ as the absorbent, demonstrated a consistent ClO_2_ release rate during the first 48 h. As shown in Figure 1e and Figure 1f, 50% gelatin and 0.3 mol/L CaCl_2_ showed the most stable release of ClO_2_. Therefore, oxalic acid and gelatin were selected as the H^+^ provider and adhesive, and CaCl_2_ was selected as the absorbent. The outcomes of the orthogonal experiments that were carried out are displayed in Table S4. The findings revealed the following order of primary components that affected ClO_2_ release: NaClO_2_ > oxalic acid > CaCl_2_ > gelatin. The preparation of the optimal ClO_2_ slow-releasing card involved the following steps: card A was soaked in 2.5 mol/L NaClO_2_ and 0.3 mol/L CaCl_2_. Card B was soaked in 1 mol/L oxalic acid and 0.3 mol/L CaCl_2_. Then, the cards were dried and adhered with 60% gelatin using a press machine.

### 3.2. Optimization of Color Indicator in ClO_2_ Slow-Releasing Card

**Figure 2:**
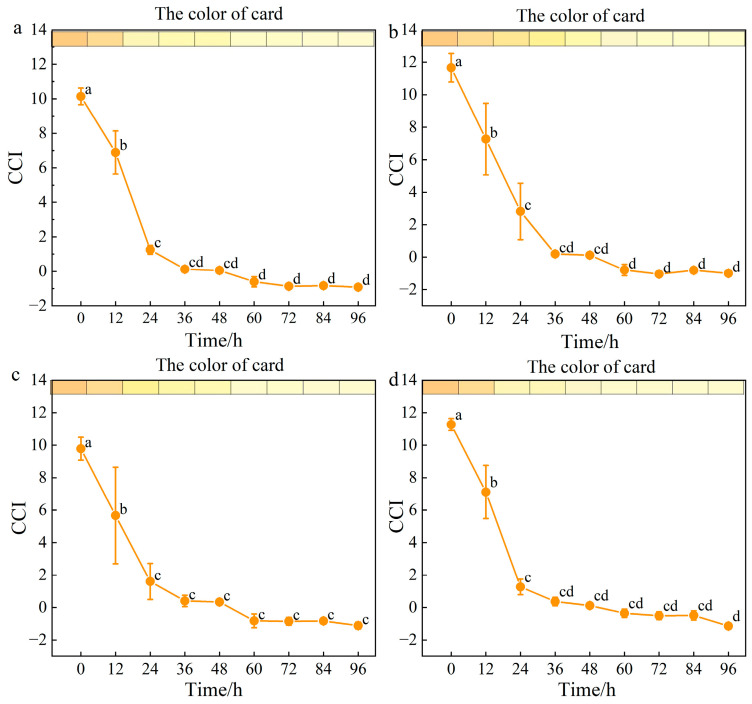
Optimization of color indicator of card. (**a**) Natural pigment; (**b**) natural pigment/β-cyclodextrin = 15:1; (**c**) natural pigment/β-cyclodextrin = 15:5; and (**d**) natural pigment/β-cyclodextrin = 15:10. Different letters represent significant (*p* < 0.05) changes between storage days.

To visualize whether ClO_2_ was released completely, the natural pigment was added into card B of the ClO_2_ slow-releasing card. As shown in Figure 2, the color of the ClO_2_ slow-releasing card changed from dark yellow to light yellow in 96 h, with the CCI of groups 1, 2, 3, 4 decreasing from 10.14, 11.66, 9.78, and 11.27 to −0.91, −1.0, −1.1, and −1.14, respectively. Notably, the CCI of group 2 (natural pigment: β-cyclodextrin = 15:1) decreased slowly at 24 h, and thereby could be the optimal color indicator of the ClO_2_ slow-releasing card. Previous research proved that β-cyclodextrin significantly enhanced the color stability of natural anthocyanin pigments. The special molecular container-like properties characterized by a hydrophilic exterior and a hydrophobic interior contributed to the stabilization of the pigments [21,22].

### 3.3. Stability Evaluation of ClO_2_ Slow-Releasing Card

**Figure 3:**
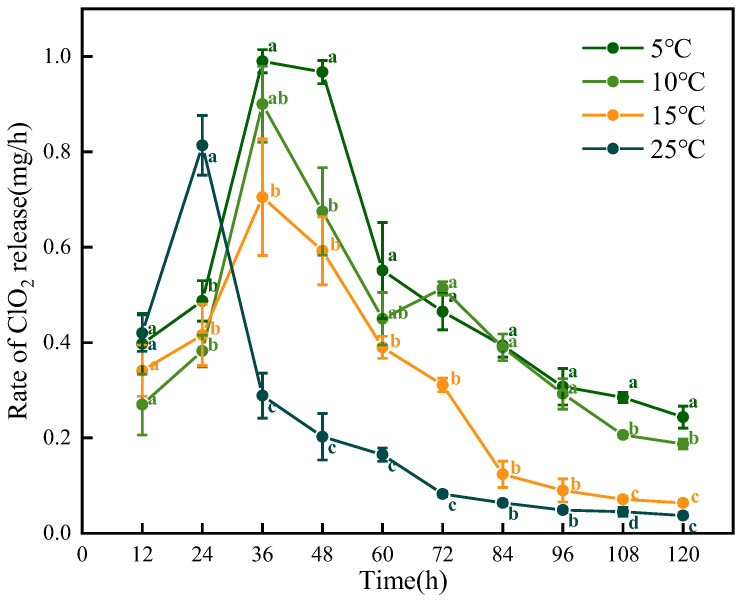
Effect of different temperatures on the release rate of ClO_2_. Different letters represent significant (*p* < 0.05) changes between groups on the same day.

Temperature significantly influences the rate and outcome of chemical reactions [23]. The optimized ClO_2_ slow-releasing card with a color indicator was used at 5 to 25 °C to verify its stability (Figure 3). The ClO_2_ slow-releasing card exhibited a maximum release time exceeding 120 h at temperatures ranging from 5 °C to 25 °C, with the peak release rate consistently occurring within 24–48 h. Notably, an increase in temperature led to earlier emergence of the ClO_2_ release peak. Interestingly, there were two release peaks when the card was used at 5 °C. The ClO_2_ release rates were 0.99 mg/h at 36 h and 0.97 mg/h at 48 h. Previous studies have established that the shelf life of litchi typically ranges from 3 to 10 days at 5–25 °C [10,24]. The pericarp discoloration and browning of litchi generally started within 12–24 h after harvesting [25,26]. The ClO_2_ slow-releasing card developed in this study exhibited a peak release within the first 24 to 48 h and maintained a stable release until 120 h. As a result, it could be a safe, effective, and economical solution for postharvest litchi.

### 3.4. Evaluation of ClO_2_ Slow-Releasing Card on Litchi Basic Qualities

**Figure 4:**
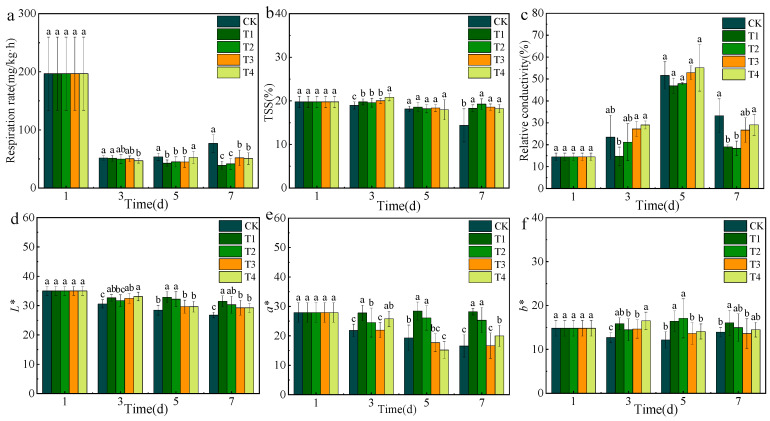
Effect of ClO_2_ slow-releasing card on respiration rate (**a**), total soluble solids (**b**), relative electrical conductivity (**c**), L* (**d**), a* (**e**), and b* (**f**) of litchi during storage. Different letters represent significant (*p* < 0.05) changes between groups on the same day.

No pieces (CK), half a piece (T1), one piece (T2), two pieces (T3), and three pieces (T4) of slow-releasing cards were placed in a sealed box with 3 kg litchi, respectively. The basic qualities of the litchi, the including respiration rate, total soluble solids, and relative conductivity, were compared. Postharvest litchi primarily undergoes respiration as its main physiological metabolic activity, which subsequently consumes nutrients from the fruit, ultimately resulting in a decline in fruit quality [27]. As shown in Figure 4a, the respiration rates of the treatment groups were 49.1%, 45.82%, 32.16%, and 33.92% lower than that of the CK group on the 7th day. Particularly, the respiration rate of litchi in the T1 group was 39.05 mg/(kg·h) at 7 d, showing the lowest respiration rate. The respiration rate of CK exhibited a sudden increase due to spoilage of the litchi on the last day. This indicated that the respiration rates of litchi were significantly inhibited by ClO_2_. Research has suggested that ClO_2_ could interfere with electron transport within the mitochondria, specifically by suppressing the enzyme cyclooxygenase during the process of aerobic respiration [28].

Total soluble solids (TSS) serve as crucial flavor indicators of fruit, and a higher content of TTS is related to superior taste [27]. As shown in Figure 4b, the TSS of the CK group exhibited a decreasing trend from the 1st day to the 7th day, with values of 19.76%, 18.95%, 18.15%, and 14.37%. The TSS of the ClO_2_ treatment groups was significantly (*p* < 0.05) higher than that of the CK group by 21.4%, 25.4%, 22.6%, and 21.1% on the 7th day, respectively. Our findings showed that the TSS of litchi was significantly (*p* < 0.05) improved and the respiration rate was decreased using the ClO_2_ slow-releasing card. Previous studies demonstrated that the persistent utilization of sugar as a substrate during fruit respiration is a primary factor contributing to the reduction in TSS [29,30].

Relative conductivity is a sensitive indicator of cell integrity. An increase in relative conductivity indicates increased plasma membrane permeability and gradual disruption of cellular compartmentation [27]. As shown in Figure 4c, the relative conductivity of the treatment groups and the CK group were 19.1%, 18.4%, 26.7%, 29.1%, and 33.21% on the 7th day. Litchi of T1 maintained the lowest relative conductivity, indicating the least damage to cell integrity. Three crucial quality analyses revealed that litchi of T1 had superior qualities, with the lowest respiration rate and relative conductivity, and highest TSS.

### 3.5. Evaluation of Effect of ClO_2_ Slow-Releasing Card on Litchi Surface Color

The surface color of litchi not only reflects changes in freshness and maturity, but also plays a crucial role in influencing consumer acceptance [27]. The values of L*, a*, and b* are a direct reflection of fruit browning [31]. As shown in Figure 4d, a gradual decrease was observed in the L* values of litchi in all groups, indicating the rapid occurrence of pericarp browning in litchi. In this study, the L* values of T1, T2, T3, and T4 exhibited a notable increase (*p* < 0.05) compared to that of CK on the 7th day, with the values rising by 15.03%, 11.82%, 8.64%, and 8.48%, respectively. Similarly, the a* values of the four treatment groups increased by 42.2%, 34.54%, 0.6%, and 17.12%, respectively, compared with that of CK (Figure 4e). Among all treatment groups, the values of L*, a*, and b* of T1 were highest on the 7th day, indicating the best surface color and lowest pericarp browning.

### 3.6. Evaluation of ClO_2_ Slow-Releasing Card on Litchi Anthocyanins

**Figure 5:**
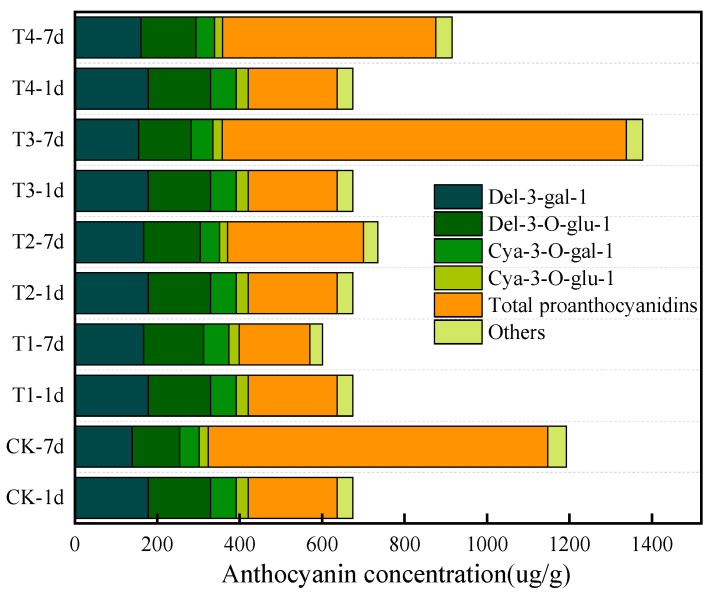
Anthocyanin concentration of litchi pericarp in CK, T1, T2, T3, and T4 groups. Others include Pet-3-O-glu-1, Peo-3-gal-1, Peo-3-O-glu-1, Cya-3-ara-1, Del-1, Peo-3-ara-1, Cya-1, Pet-1, Peo-1, and Mal-1. Total proanthocyanidins include Pro-A1-1, Pro-A2-1, Pro-B1-1, Pro-B2-1, Pro-C1-1, Pro-B3-1, and Pro-B4-1.

Anthocyanins, a subclass of flavonoids, are responsible for the red coloration of litchi pericarp [32,33,34]. The degradation of anthocyanins can result in the browning of litchi [32]. As shown in Figure 5, 21 anthocyanins were identified by ultra-high-performance liquid chromatography–tandem mass spectrometry (UPLC-MS.MS). Del-3-gal-1, Del-3-O-glu-1, Cya-3-O-gal-1, and Cya-3-O-glu-1 accounted for 62.83% of the total anthocyanin content, and were the predominant anthocyanins in litchi pericarp. Four predominant anthocyanins of all groups decreased sharply on the 7th day. Except for T1, the total anthocyanins of all groups increased on the 7th day of storage compared with the 1st day. Interestingly, the total proanthocyanidins of T1 decreased on the 7th day, whereas those of the other groups increased. Proanthocyanidins are colorless polymers of flavonoids and important secondary metabolites in plants formed by the condensation of flavan-3-ol units [35,36,37]. The accumulation of proanthocyanidins can boost plants’ defenses against various stresses like temperature extremes, drought, physical harm, UV rays, and fungal diseases [38,39,40]. We speculated that four predominant anthocyanins of T1 were stable, and little metabolic stress arose due to the relatively stable TSS, respiration rate, and relative conductivity, and therefore, the fewest proanthocyanidins were consumed.

### 3.7. Correlation Analysis of Litchi Color Indicators with Key Anthocyanins

**Figure 6:**
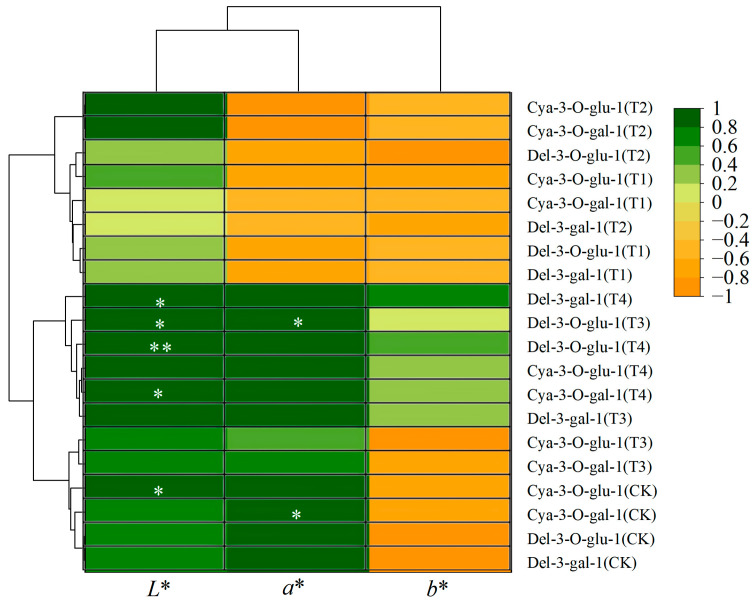
Correlation between color indicators and key anthocyanins of litchi. Green represents positive correlation, yellow represents negative correlation, * represents *p* < 0.05, ** represents *p* < 0.01.

To investigate the relationship between litchi color changes and anthocyanins, correlation analyses between L*, a*, b*, and the four predominant anthocyanins were performed. As shown in Figure 6, the correlation analyses between L*, a*, b*, and the four predominant anthocyanins revealed a significant positive correlation (R^2^ = 0.971) between Cya-3-O-gal-1 and the a* in CK, indicating a sharp decline in Cya-3-O-gal-1 and strong pericarp browning in CK. On the contrary, Cya-3-O-gal-1 in the T1 group slightly decreased by 2.09% from the 1st day to the 7th day of storage, showing no significant positive correlation with a* and L*. Previous studies reported that the product derived from anthocyanidin degradation possesses a structure akin to catechol, which serves as an effective substrate for polyphenol oxidase. Consequently, this similarity can expedite the enzymatic browning reaction catalyzed by polyphenol oxidase [41,42]. We speculated that the low-releasing ClO_2_ of T1 effectively inhibited the degradation of anthocyanins, especially Cya-3-O-gal-1. This could be one of the reasons for the superior pericarp color of T1.

## 4. Conclusions

In this study, a color-variable chlorine dioxide (ClO_2_) slow-releasing card was prepared and further used for postharvest litchi. The ClO_2_ releasing time of the card was more than 120 h at 5–25 °C, and it could change color from dark yellow to white. The qualities of 3 kg litchi treated with no pieces (CK), half a piece (T1), one piece (T2), two pieces (T3), and three pieces (T4) of ClO_2_ slow-releasing card were compared. Results showed that litchi of T1 had significantly (*p* < 0.05) higher L* and a* values but a lower respiration rate and relative conductivity than CK after 7 days of storage, showing the best pulp qualities and pericarp color. Further correlation analyses between L*, a*, b*, and the four predominant anthocyanins revealed a significant positive correlation (R^2^ = 0.971) between Cya-3-O-gal-1 and the a* in CK, indicating a sharp decline in Cya-3-O-gal-1 and strong pericarp browning in CK. On the contrary, the low-releasing ClO_2_ of T1 effectively inhibited Cya-3-O-gal-1 degradation. This could be one of the reasons for the superior pericarp color of T1. This study provides a visual, efficient, and economical solution for postharvest litchi.

## Data Availability

The original contributions presented in this study are included in the article/Supplementary Materials. Further inquiries can be directed to the corresponding authors.

## Supplementary Materials

The following supporting information can be downloaded at: https://www.mdpi.com/article/10.3390/foods14010136/s1, Table S1: Optimization of ClO_2_ slow-releasing card components by L16(4^4^) orthogonal experiment; Table S2: Mobile phase gradient of UPLC; Table S3: Mass spectrometry information of 21 anthocyanins; Table S4: Results of orthogonal experiment.
