# Supplementary material for: Development and Assessment of a Color-Variable Chlorine Dioxide Slow-Releasing Card for Litchi Preservation
Source: Foods. 2025 Jan 6;14(1):136. doi: 10.3390/foods14010136 (PMC11720410; doi:10.3390/foods14010136)
Supplement: Supplementary file 1 [file foods-14-00136-s001.zip › foods-3389213-supplementary.pdf]

Table S1. Optimization of ClO_2 slow-releasing card components by $L_{16}(4^4)$ orthogonal experiment

Group	$NaClO_2$ (mol/L)	Oxalic acid (mol/L)	Gelatin (%)	$CaCl_2$ (mol/L)
1	1	0.5	30	0.1
2	1	1	40	0.2
3	1	1.5	50	0.3
4	1	2	60	0.4
5	1.5	0.5	40	0.3
6	1.5	1	50	0.4
7	1.5	1.5	60	0.1
8	1.5	2	30	0.2
9	2	0.5	50	0.4
10	2	1	60	0.1
11	2	1.5	30	0.2
12	2	2	40	0.3
13	2.5	0.5	60	0.1
14	2.5	1	30	0.2
15	2.5	1.5	40	0.3
16	2.5	2	50	0.4

Table S2. The mobile phase gradient of UPLC

Time (min)	A: 0.05% formic acid aqueous solution (%)	B: acetonitrile (%)
0	10	90
3	90	10
3.5	10	90
5	10	90

Table S3. Mass spectrometry information of 21 anthocyanins

Number	Name	Abbreviation	Molecular formula	Molecular mass	Parent ion(m/z)	Ion pair(m/z)	De-clustering potential(ev)	Collision energy(ev)	Ion mode
1	Delphinidin-3-galactoside chloride	Del-3-gal	$C_{21}H_{21}ClO_{12}$	500.8372	465.4	303/228.9	100	23/63	+
2	Delphinidin-3-O-glucoside chloride	Del-3-O-glu	$C_{21}H_{21}ClO_{12}$	500.8400	465.4	303.1/228.8	90	26/74	+
3	Cyanidin-3-O-galactoside chloride	Cya-3-O-gal	$C_{21}H_{21}ClO_{11}$	484.8400	449.3	287.1/137	90	25/67	+
4	Cyanidin-3-O-glucoside chloride	Cya-3-O-glu	$C_{21}H_{21}ClO_{11}$	484.8400	449	287/213.1	90	25/71	+
5	Petunidin-3-O-glucoside chloride	Pet-3-O-glu	$C_{22}H_{23}ClO_{12}$	514.8600	479.4	317.1/302.1	90	26/56	+
6	Peonidin 3-galactoside chloride	Peo-3-gal	$C_{22}H_{23}O_{11}Cl$	498.8644	463.4	300.9/286	100	30/55	+
7	Peonidin-3-O-glucoside chloride	Peo-3-O-glu	$C_{22}H_{23}ClO_{11}$	498.8600	463.2	285.9/300.8	80	53/75	+
8	Cyanidin-3-arabinoside chloride	Cya-3-ara	$C_{20}H_{19}ClO_{10}$	454.8119	418.9	287.1/365.1	180	24/16	+
9	Delphinidin chloride	Del	$C_{15}H_{11}ClO_{7}$	338.7000	303.4	229.2/256.8	170	44/34	+
10	Peonidin-3-arabinoside chloride	Peo-3-ara	$C_{21}H_{21}ClO_{10}$	468.8400	434.9	303.1/229.1	100	22/61	+
11	Cyanidin chloride	Cya	$C_{15}H_{11}ClO_{6}$	322.7000	286.9	137.2/212.8	150	38/43	+
12	Petunidin chloride	Pet	$C_{16}H_{13}ClO_{7}$	352.7200	317	245.1/203	100	48/50	+
13	Peonidin chloride	Poe	$C_{16}H_{13}ClO_{6}$	336.7200	301.4	286.2/201.1	180	33/45	+
14	Malvidin chloride	Mal	$C_{17}H_{15}ClO_{7}$	366.7500	331	314.9/241.7	150	38/40	+
15	Procyanidin A1	Pro-A1	$C_{30}H_{24}O_{12}$	576.5	575	288.9/449	-100	‘-32/-32	-
16	Procyanidin A2	Pro-A2	$C_{30}H_{24}O_{12}$	576.5	575	449/288.8	-100	’-30/-35	-
17	Procyanidin B1	Pro-B1	$C_{30}H_{26}O_{12}$	578.52	577.1	289.1/407	-100	‘-32/-32	-
18	Procyanidin B2	Pro-B2	$C_{30}H_{26}O_{12}$	578.52	577	288.9/406.8	-100	‘-35/-28	-
19	Procyanidin C1	Pro-C1	$C_{45}H_{38}O_{18}$	866.77	865.3	577.1/406.8	-100	‘-30/-52	-
20	Procyanidin B3	Pro-B3	$C_{30}H_{26}O_{12}$	578.52	577.1	289.1/407.1	-100	‘-30/-30	-
21	Procyanidin B4	Pro-B4	$C_{30}H_{26}O_{12}$	578.52	577.1	288.9/406.9	-100	‘-35/-32	-

Table S4. The results of orthogonal experiment

Group	$NaClO_2$(mol/L)	Oxalic acid(mol/L)	Gelatin (%)	$CaCl_2$(mol/L)	The total release amount of ClO_2(mg)
1	1	1	1	1	16.47
2	1	2	2	2	19.80
3	1	3	3	3	26.63
4	1	4	4	4	23.43
5	2	1	2	3	20.97
6	2	2	3	4	36.17
7	2	3	4	1	33.87
8	2	4	1	2	24.50
9	3	1	3	4	20.73
10	3	2	4	1	35.40
11	3	3	1	2	31.53
12	3	4	2	3	39.37
13	4	1	4	1	29.37
14	4	2	1	2	42.33
15	4	3	2	3	38.37
16	4	4	3	4	31.03
K1	86.33	87.53	114.83	115.10	
K2	115.50	133.70	118.50	118.17	
K3	127.03	130.40	114.57	125.33	
K4	141.1	118.33	122.07	111.37	
k1	28.78	29.18	38.28	38.37	
k2	38.50	44.57	39.5	39.39	
k3	42.34	43.47	38.19	41.78	
k4	47.03	39.44	40.69	37.12	
R	18.26	15.39	2.50	4.66	
